# Beyond Graphene Anode Materials for Emerging Metal Ion Batteries and Supercapacitors

**DOI:** 10.1007/s40820-018-0224-2

**Published:** 2018-10-09

**Authors:** Santanu Mukherjee, Zhongkan Ren, Gurpreet Singh

**Affiliations:** Department of Mechanical and Nuclear Engineering, Kansas State University, Manhattan, KS 66506 USA

**Keywords:** Two-dimensional materials, Transition metal dichalcogenides, MXene, Exfoliation, Top-down, Anodes

## Abstract

Intensive research effort is currently focused on the development of efficient, reliable, and environmentally safe electrochemical energy storage systems due to  the ever-increasing global energy storage demand. Li ion battery systems have been used as the primary energy storage device over the last three decades. However, low abundance and uneven distribution of lithium and cobalt in the earth crust and the associated cost of these materials, have resulted in a concerted effort to develop beyond lithium electrochemical storage systems. In the case of non-Li ion rechargeable systems, the development of electrode materials is a significant challenge, considering the larger ionic size of the metal-ions and slower kinetics. Two-dimensional (2D) materials, such as graphene, transition metal dichalcogenides, MXenes and phosphorene, have garnered significant attention recently due to their multi-faceted advantageous properties: large surface areas, high electrical and thermal conductivity, mechanical strength, etc. Consequently, the study of 2D materials as negative electrodes is of notable importance as emerging non-Li battery systems continue to generate increasing attention. Among these interesting materials, graphene has already been extensively studied and reviewed, hence this report focuses on 2D materials beyond graphene for emerging non-Li systems. We provide a comparative analysis of 2D material chemistry, structure, and performance parameters as anode materials in rechargeable batteries and supercapacitors.
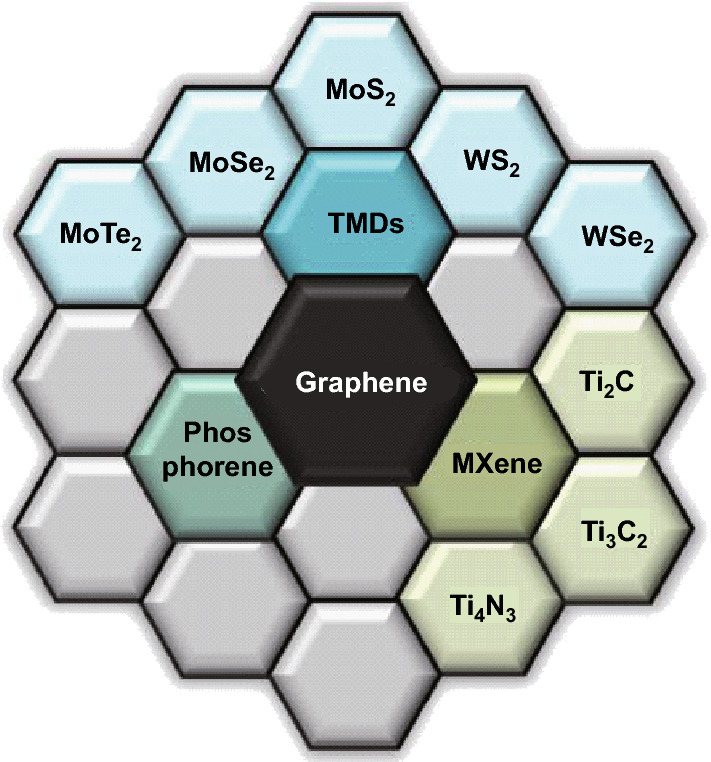

## Highlights

- A comprehensive review of novel 2D materials, particularly MXene and phosphorene, for applications as electrodes in both batteries and supercapacitors. The performance of these materials, in addition to their fabrication techniques, structure, and electrochemistry, is highlighted.
- Separate sections are dedicated to explaining the limitations of conventional anode materials and the role of 2D materials in addressing these deficiencies, in addition to the future path of development of this field of research.

## Introduction

LIBs have been the predominant rechargeable metal ion battery system for energy storage ever since they were first introduced commercially by Sony in the early 1990s [1]. They have become ubiquitous in their applications, which include smartphones and tablets, and even battery-powered vehicles [2, 3]. It is not an exaggeration to suggest that LIBs have played a leading role in revolutionizing the field of consumer electronics. There are several reasons for the excellent performance of LIBs; the relatively small ionic size of Li^+^ (0.76 Å) and the consequent ease of intercalation into host lattices, the large energy and power densities and the low reduction potential (− 3.04 V vs standard hydrogen electrode) [4, 5] are a few examples. Regardless of these advantages, LIBs are hindered by several important deficiencies. The elevated reactivity of Li metal, the flammability of organic electrolytes, the low quantity of raw material (Li) in the earth’s crust leading to a high cost of Li procurement and other issues related to the disposal of spent cells are some of the major concerns that are posed by current LIB systems [1, 6].

With this perspective in mind, there has been a dedicated effort to develop viable alternatives to LIBs in recent years. A few examples of notable non-LIB metal ion rechargeable battery systems that have received significant attention include the sodium ion battery system (SIB), the potassium ion battery system (KIB), the magnesium ion battery system (MIB) along with aluminum and calcium ion battery systems [7, 8]. These materials are significantly more abundant in the earth’s crust than Li, and their disposal does not pose as much of an environmental hazard. Consequently, they are being proposed for applications in medium- to large-scale grid storage systems [5, 9]. However, the large size of these ions (Na^+^, K^+^ etc.) often results in challenges during intercalation, lower ionic mobilities, and poor kinetics [4, 10]. For example, Na^+^ ion cannot intercalate easily into graphite (the standard anode material for LIB systems) due to its large size and energetic considerations [11]. Therefore, the careful reconfiguration of the microstructure and efficacious engineering of the electrode design is of considerable significance. Table 1 provides a comparative analysis of the important aspects associated with the different types of metal ions for emerging rechargeable electrochemical storage devices with respect to lithium.

**Table 1:** Comparison of the critical parameters of Li with other important metal ions that are used in emerging metal ion rechargeable electrochemical storage devices. These parameters play an important role in determining the choice of raw material

Important parameters	Li^+^	Na^+^	K^+^	Mg^2+^	Ca^2+^	References
Ionic structure						
Ionic (Shannon) radii (Å)	0.76	1.02	1.38	0.72	1.00	[4]
Relative atomic mass	6.94	23.00	39.10	24.31	40.07	[5]
Abundance in earth crust (wt%)	0.0017	2.6	2.4	1.9	3.4	[5, 9]
Atomic density (g cm^−3^)	0.534	0.968	0.89	1.738	1.55	*
*E*_0_ versus SHE (V)	− 3.04	− 2.71	− 2.93	− 2.70	− 3.80	[5]
Theoretical capacity						
(mAh g^−1^)	3861	1166	685	2205	1340	[5, 9]
(mAh cm^−3^)	1378	1193	1059	1085	987	[5]
Cost of industrial grade metal (US$/ton)	130,000	3200	14,000	2300	3300	**

*J.A. Dean (ed), Lange’s Handbook of Chemistry (15th Edition), McGraw-Hill, 1999; Section 3; Table 3.2 Physical constants of inorganic compounds

**http://original.metal.com

**Fig. 1:**
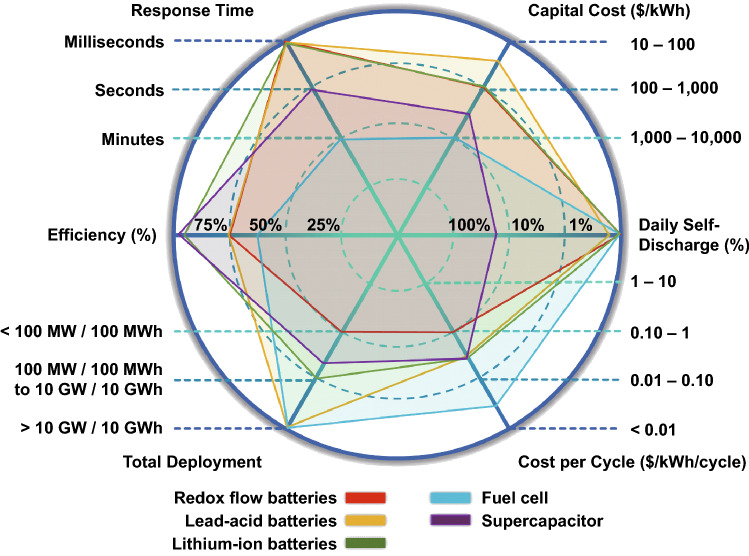
Spider graph representation of critical parameters of the different electrochemical energy storage systems. Data for the figure have been obtained from Ref. [12]

A schematic representation of the different types of conventional electrochemical energy storage systems, the economics involved, and their performance criteria are provided in Fig. 1. It is noted that LIBs provide significantly low self-discharge characteristics and high efficiency compared to other electrochemical energy storage systems. Therefore, to match the best performance of LIBs and to improve upon those aspects where they are inferior, the development and understanding of novel anode materials is necessary. Hence, novel 2D materials are investigated. Based on their properties, it is expected that 2D materials will be able to enhance the performance of non-Li metal ion rechargeable batteries.

2D materials have recently received widespread attention, especially for applications in electronic devices, electrochemical energy storage (batteries and supercapacitors), anti-corrosion applications, photonics, etc. [5]. These materials present layered structures with a dimensional constraint in one direction [5, 13]. This layered structure provides a large surface area which makes them attractive for applications as negative electrodes in electrochemical energy storage systems [13].

**Fig. 2:**
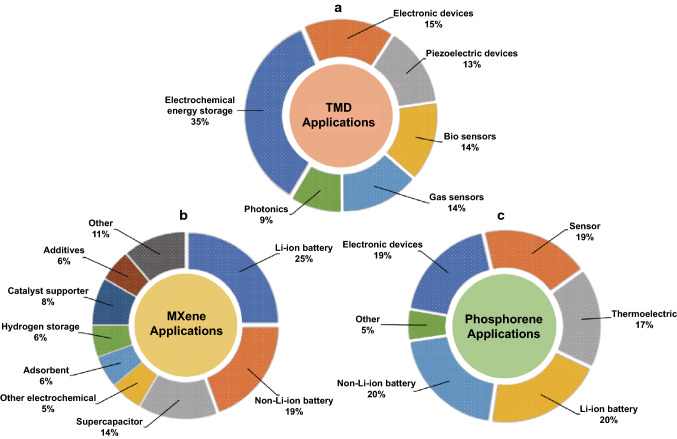
Statistical analysis of the application of beyond graphene 2D materials: **a**–**c** Pie chart analysis showing the weight of the various applications of TMDs, MXene, and phosphorene, respectively. Electrochemical energy storage occupies an important role in the application regimes of each of the 2-D materials, which is indicative of the current trend of their applications in batteries and supercapacitors

*Graphene* is a single sheet of graphite and is one of the most popular 2D materials [13, 14]. The popularity of this material stems from its diverse range of favorable properties; large intrinsic carrier mobility [~ 200,000 cm^2^ (Vs)^−1^], high mechanical strength (Young’s modulus of ~ 1 TPa), very high electrical conductivity (~ 106 S cm^−1^), and high theoretical specific surface area (2630 m^2^ g^−1^), as examples [15–17]. A large number of diverse studies have been performed on graphene with an emphasis on potential applications ranging from electrochemistry to solar cells, based on these superior properties [14, 18]. *Transition metal dichalcogenides* (*TMDs*), e.g., MoS_2_, MoSe_2_, WS_2_, WSe_2_, are a class of compounds with layered structures that have demonstrated promise for electrochemical energy storage and other applications including as catalysis, sensors, piezoelectric devices [19, 20]. *MXenes* are 2D inorganic compounds (usually transition metal nitrides or carbides) that continue to generate considerable interest [21]. They possess good conductivity and are being investigated as anodes for battery and supercapacitor applications as well as for other areas such as hydrogen storage and as adsorbents [22, 23]. *Phosphorene* is single layer/few layers of black phosphorus (BP), which is regarded as another novel 2D material with interesting properties [24]. Phosphorene possesses a hexagonal lattice structure which resembles graphene and has been targeted for applications in electrochemical energy storage, solar cells, sensors, etc. [25–28]. Figure 2 illustrates a detailed diagrammatic representation including a statistical analysis of the different areas of application of 2D-layered materials beyond graphene.

A large number of reports on the application of graphene as an electrode material for electrochemical energy storage devices already exist in the scientific literature [29, 30]. Therefore, as a forward-looking review, this article will focus on the analysis and review of 2D materials beyond graphene in emerging non-Li rechargeable electrochemical energy storage systems.

## Current State-of-the-art and 2D Materials as Anodes for Emerging Metal Ion Rechargeable Battery Systems

The current state-of-the-art anodes for non-Li rechargeable metal ion battery systems consist of a diverse class of compounds. The subsequent paragraph will provide a brief discussion of these compounds and detail how 2D materials can be used to address their deficiencies.

Initially, different carbonaceous materials, e.g., graphite, hard carbons, were considered for application as anodes. However, the storage of Na^+^ ions in graphite is unsuitable due to the lack of sufficient host sites and the unfavorable energetics, and the storage capacity of sodium in graphite is consequently only 35 mAh g^−1^ [9]. Potassium intercalates into graphite unlike sodium; however, large voltage hysteresis is prevalent and the rate capabilities are relatively poor at high C rates [31].

Metal oxides are another important class of compounds that have been investigated for this purpose. Oxides such as Fe_2_O_3_, CuO have a high theoretical storage capacity for sodium. However, they undergo large volume changes due to a conversion reaction which reduces their lifespan and thereby limits their utility in this regard [9]. NASICON-type anodes, e.g., NaTi_2_(PO_4_)_3_, provide a potential solution to this problem with their high structural stability and good ionic conductivity [32]. However, they exhibit poor electronic conductivities and are relegated to being used primarily as composites [32]. Composite metal oxides such as Co_3_O_4_–Fe_2_O_3_ have shown some promise in KIB systems with demonstrated capacities of 350 mAh g^−1^ [33]. Low-strain transition metal oxides such as Li_4_Ti_5_O_12_ for MIB systems have also been investigated and have demonstrated good cycling abilities, but a more thorough evaluation is necessary [34].

Alloy-based anodes (alloys of Sn, Sb, etc.) have been investigated as potential anodes for SIB systems. In particular, Sn-based alloys provide a theoretical capacity as high as 790 mAh g^−1^. However, the potential application of these alloys is restricted due to their large volume expansion (~ 520% for some Sn alloys) as a result of the alloying/intercalation process. As a result, stability issues are often encountered [35]. Phosphorus has been used as an alloying anode material for KIB systems because of its large theoretical capacity (843 mAh g^−1^). However, the issue of large volume expansion (~ 190%) is also prevalent and causes rapid capacity fading [36]. Metal alloys, e.g., Bi-Sb, have also been investigated as anodes in MIB systems because of their high theoretical capacity (~ 2205 mAh g^−1^). However, in this case, the problem lies in developing a better understanding of the electrode–electrolyte interface because conventional anodes are unsuitable in the electrolyte of choice [37].

2D materials with their unique properties are well suited to overcome the limitations of conventional anode systems. These materials can be fabricated with relative ease by liquid-based exfoliation techniques with a high degree of scalability [13]. Because the exfoliation process does not typically involve any chemical reactions, the resulting 2D materials are able to retain their morphology and chemical structure [13]. These materials have a large surface area due to their morphology, enhanced kinetics and high theoretical capacities. One of the most important aspects of 2D materials is that their interlayer spacing can be manipulated by pre-intercalating guest species (e.g., metal ions, polymers etc.) inside their layers during the fabrication process [38, 39]. For example, in TMDs, the rather large interlayer spacing (e.g., 0.65 nm for MoS_2_) provides a rather conducive environment for accommodating large-sized alkali metal ions such as Na^+^, K^+^ [5]. MXene layers/sheets have also demonstrated excellent electrical conductivity and efficient sodium storage capacity [5]. Phosphorene has also demonstrated high theoretical capacities and is more stable unlike its bulk precursor, black phosphorus, which undergoes large volume changes during interaction with intercalating ions [40].

## Structure and Electronic Properties of 2D Materials

The crystal structure and electronic properties of 2D materials are important aspects that determine their overall electrochemical performance and therefore merit a detailed overview. Also, the properties of these materials in their single-/few-layered forms are different from those of their bulk morphology. This aspect plays an important role in influencing their performance as electrodes [41]. The following subsections discuss these properties in greater detail.

### TMDs

TMDs usually demonstrate the empirical chemical formula given by MX_2_, where M is a transition metal (Mo, W etc.) and X is a chalcogen atom (S, Se, etc.) [42]. Consequently, a wide variety of TMDs exists with different fundamental properties: from the semiconducting MoS_2_ to the semi-metal WTe_2_ [41, 43]. The TMDs from groups IV–VII usually demonstrate a layered morphology that is generally presented as a crystal structure resembling graphene [44, 45]. It has been observed that the individual TMD layers have a thickness in the range of approximately 6–7 Å [44]. Since the layers in TMDs are held together by weak van der Waals forces of attraction, they serve as prime candidates for being subjected to exfoliation when appropriate techniques are applied [46].

In the MX_2_ lattice, the transition metal and the chalcogen atom exhibit oxidation states of + 4 and − 2, respectively [41]. Polymorphism exists in the few-layered form more commonly than in the bulk form. Some common polymorphs include the 1T, 2H, and 3R phases, respectively, where the letters indicate the trigonal, hexagonal, and rhombohedral orientations, respectively [41, 47]. However, for single-layered TMDs, the only two polymorphs possible are the trigonal and octahedral phases, respectively. Generally, the electronic structure of the transition metal (*d*-orbital) determines the polymorphic orientation, e.g., TMDs with group IV transition metals (*d*^0^) usually crystallize in the octahedral phase, whereas those with group V transition metals (*d*^1^) exhibit both octahedral and trigonal phases [41].

### MXene

MXenes generally demonstrate the composition *M*_*n*+1_*X*_*n*_*T*_*x*_, where M is an early transition metal (Ti, Zr, Hf, V, Nb, etc.), *X* is *C* or *N* and *n* = 1, 2 or 3, and T is a surface termination group (oxygen, fluorine or hydroxyl) [48]. These MXenes are generally obtained by selective leaching of the A phases out of their precursor MAX phases. The latter is a large family (approximately 70) of ternary carbides and nitrides that usually occur in layered form interspersed with group 13 and/or 14 elements separating the layers [49, 50]. Structurally, these MAX phases are alternate stacks of hexagonal MX layers with the layers of the A atoms existing in a densely packed fashion along the c-axis [51]. The type of bonding in these MAX phases is a hybrid of covalent, metallic and ionic [51].

Since the MXenes are obtained from their parent MAX phases, the M atoms are similarly arranged in a close-packed fashion with the X atoms in the octahedral interstices [52]. Generally, the MXenes exhibit a hexagonal close-packed (hcp) structure and are represented by formulas of the type M_3_C_2_ (e.g., Ti_3_C_2_), M_4_C_3_ (e.g., Nb_4_C_3_), etc. Also, since the MXenes are synthesized by etching their precursor MAX phases, they contain the respective –OH, –O, and/or –F termination groups [48, 52]. The results of neutron scattering experiments have indicated that van der Waals bonds exist between the sheets together with hydrogen bonding between the O or OH groups within a sheet and the F group of an adjacent sheet [53]. It should also be noted the fabrication process (etching, type of etchants) strongly influences the crystallinity and the presence of defects in the final MXene product [48, 53].

### Phosphorene

Phosphorene is essentially single/few layers of bulk black phosphorus and exhibits a characteristic hexagonal (“puckered honeycomb”) structure [24]. The few layers are held together by weak van der Waals attractive forces. A slight variation is noticed between the lattice parameters of bulk black phosphorus (*a* = 3.308 Å, *b* = 4.627 Å, and *c* = 11.099 Å) and monolayer phosphorene (*a* = 3.35 Å and 4.62 Å) [24, 54]. The crystal structure of phosphorene is orthorhombic, and the P atom forms covalent bonds with three nearest neighbors [55]. Phosphorene demonstrates considerable anisotropy, and the stacking of its individual layers produces two orientations: the armchair and the zigzag [56].

Electronically, phosphorene behaves as a direct band-gap semiconductor. However, the application of strain may alter its semiconducting properties [57]. The number of layers making up the phosphorene sheets also influences its electronic properties [57]. Phosphorene also demonstrates anisotropic thermal properties; zigzag orientation produces size-dependent thermal behavior, while size does not play a role in the thermal behavior of the armchair oriented phosphorene [58, 59]. The surface properties of phosphorene can be tuned to exploit specific properties [60]. For example, Srivastava et al. demonstrated that a monovacancy defect causes the phosphorene atom to be magnetic whereas such an effect is not observed for divacancy defects [60, 61].

**Fig. 3:**
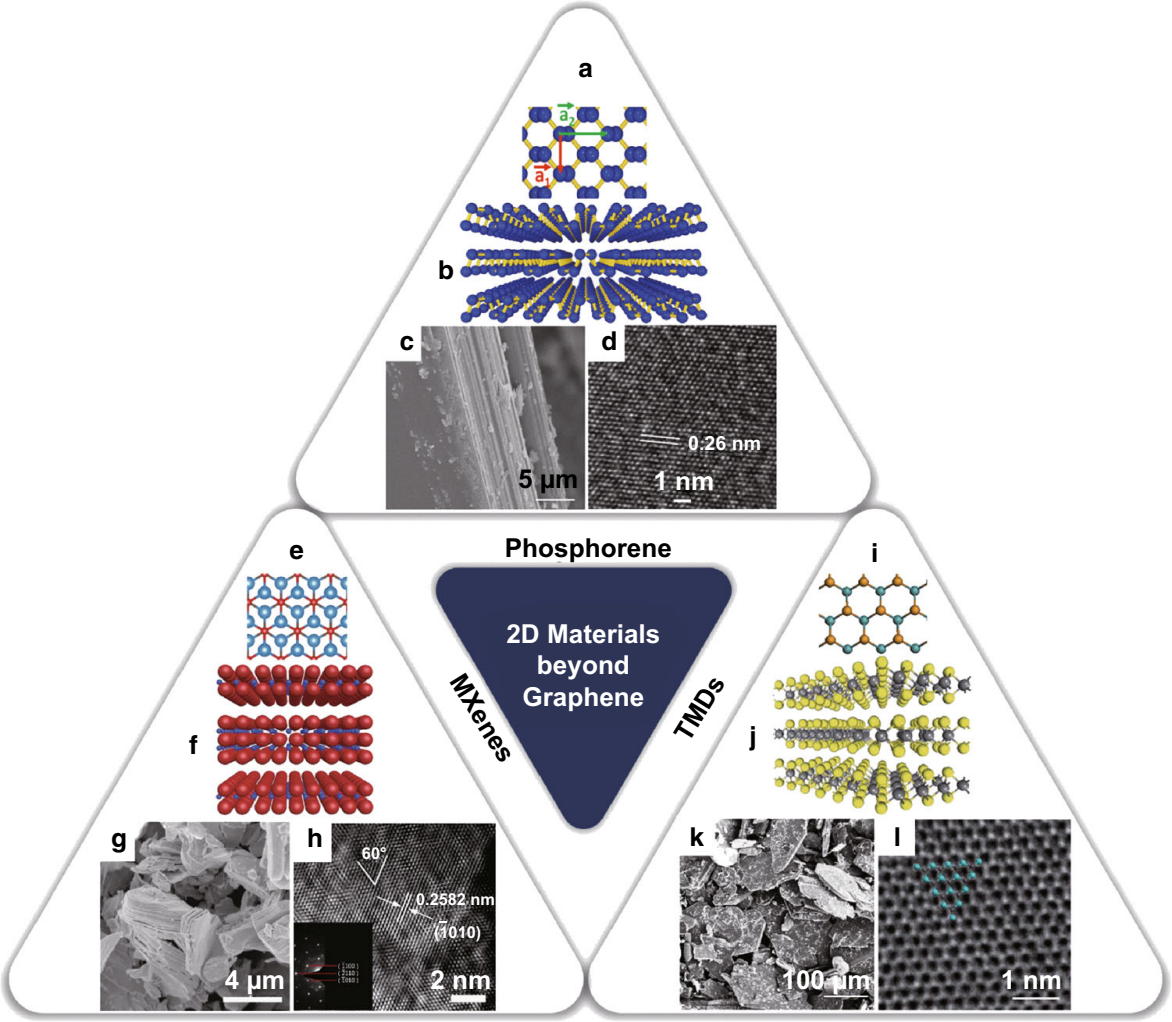
Schematic representation of structure and atomic arrangement of the layered “beyond graphene” materials which are the focus of the present work: **a**, **e**, **i** Top view of the ball and stick atomic models of phosphorene, MXene, and TMD (MoSe_2_), respectively. **b**, **f**, **j** Side view of the atomic models showing the layered structure of phosphorene, MXene, and TMD (MoSe_2_), respectively. SEM images showing the characteristic layered structure of **c**, **g**, **k** SEM images showing the characteristic layered structure of phosphorene, MXene, and TMD (MoSe_2_), respectively. **d**, **h**, **i** HRTEM image demonstrating the arrangement of the atoms in the lattice for phosphorene, MXene, and TMD (MoSe_2_), respectively [24, 62–71]. Permission allowed by copyright owners

Schematic representations of the 2D materials discussed in this section together with their crystal structures, lattice parameters, and characteristic SEM and TEM images are shown in Fig. 3.

## Fabrication of 2D Materials Relevant to Electrochemical Energy Storage Applications

The fabrication techniques of 2D materials are important since they greatly affect their final morphology, electronic properties, and even mechanical and chemical stabilities [41, 72]. There are numerous examples of reported studies on the fabrication of 2D materials in general [73]. In this section, we provide a brief summary of the fabrication techniques that are most relevant to electrochemical energy storage.

Usually, two broad empirical approaches; “top-down” and “bottom-up’ are employed for the synthesis of 2D materials [13, 74]. “Top-down” techniques involve stripping away material from a bulk 3D precursor to arrive at the layered morphology, and “bottom-up” methods are useful for generating very thin (atomically) films and layers [74, 75].

One of the most straightforward top-down techniques that have been utilized for fabricating 2D materials is the mechanical exfoliation process. In this technique, individual sheets are obtained by shearing off the constituent layers of the bulk material using an applied mechanical force [76]. Phosphorene has been produced by this technique from its precursor, black phosphorus, as has many other 2D materials [24, 77, 78]. However, this technique is unreliable from a scalability perspective even though the quality of the sheets produced is generally of high purity [24, 79–81]. Another exfoliation technique that is more commonly employed is liquid phase exfoliation. In this method, the same shearing of the constituent sheets of the bulk materials takes place in a liquid media (alcohols, ketones, acids) coupled with sonication [82, 83]. The net effect is that the process helps to overcome the relatively weak van der Waals bonding that exists between the layers of the bulk parent material [73, 84]. Intercalation-assisted liquid phase exfoliation is a modified form of the liquid exfoliation technique in which a pre-lithiated bulk 2D crystal is used as the precursor. This technique has been successful in producing relatively defect free sheets [85, 86].

Another important top-down technique that is employed, especially for the fabrication of MXenes, is the etching process [22, 48]. The etching process is used to preferentially leach out the “A” atom from the precursor MAX phase of the corresponding MXene with HF used as the most common etchant [48, 87]. It should be noted that etching is strongly dependent on reaction kinetics and different MXenes have their own specific etching times [87]. Also, if etching is done in the presence of a metal halide instead of pure HF, the halide atoms tend to intercalate in between the MXene sheets making their exfoliation even easier [88].

Top-down techniques have been favored because of their ease of processing, low cost of materials and technique, large surface area sheets and reasonable scalability. As such, they have been widely employed for electrochemical energy storage purposes [89].

**Fig. 4:**
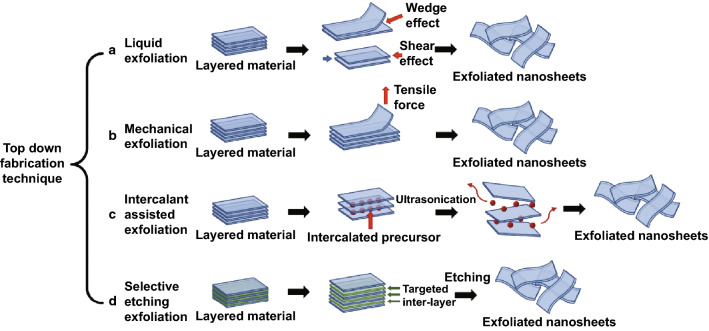
Schematic representation of various “top-down” fabrication techniques for large-scale synthesis of electrode materials: **a** Liquid exfoliation where the sheets are sheared off by sonicating the bulk material in a liquid medium. **b** Mechanical exfoliation, whereby a tensile force (possibly produced by Scotch tape) can peel off the constituent layers. **c** Intercalant-assisted exfoliation where a metal ion (usually Li^+^) is used to intercalate into the bulk precursor in a liquid media coupled with ultrasonication, which shears the sheets apart. **d** Selective etching, in which an etchant (usually strong acids, e.g., HF) is used to remove one or more constituent atoms and result in the formation of sheets

Figure 4 provides a schematic representation of the various top-down fabrication techniques.

Bottom-up techniques, e.g., chemical vapor deposition (CVD), tend to aggregate material at the most fundamental level (atoms) in a layer-wise process to produce the desired stoichiometry and morphology [90, 91]. Several bottom-up techniques have been employed in the fabrication of nanosheets; CVD and atomic layer deposition (ALD) are among the more important techniques [92, 93]. In particular, the CVD technique allows for greater control of the thickness of the final product; fabrication of the Mo_2_C MXene crystals is a good example of this capability [94, 95]. However, bottom-up techniques are not commonly used for electrochemical energy storage applications since they are costly, energy intensive and are not easily scalable processes. For the interested reader, detailed reviews of bottom-up approaches for 2D materials can be found in the literature by Ye et al. and Cai et al. [96, 97].

## Performance Review of 2D Materials as Anodes in Non-Li Metal Ion Rechargeable Electrochemical Energy Storage Devices

A discussion of the fundamental electrochemistry and the performance of the various 2D materials are discussed in the subsequent sections.

### Electrochemistry of 2D TMDs

In the case of 2D materials, the weak bonds between their constituent sheets and the ease of intercalation of alkali ions between their layers are two of the foremost properties which make them desirable as energy storage materials [98]. The bulk counterparts of these layered materials suffer from significant volume changes upon interacting with alkali metal ions in addition to low average working voltages. However, when these materials are exfoliated, they are able to accommodate a greater number of intercalating ions due to their weak out-of-plane binding [99]. Also, exfoliation increases the active surface area for electrochemical activity, reduces the tortuosity, and provides easier intercalation pathways. Among the various layered TMDs that are available, MoS_2_ are among the most studied [100]. Manipulation of the surface properties has been shown to enhance the electrochemical behavior, as has been demonstrated by Yang et al. [101]. The vertically aligned MoS_2_ nanosheets they synthesized exhibited a higher capacity, which is attributable to their dangling bonds which provided a greater active surface area for the ions traveling through the electrolyte [101]. Chowalla and coworkers have also demonstrated the ability of MoS_2_ as a promising supercapacitor electrode (specific capacitance values ranging from 400 to 700 F cm^−3^) [102]. The authors have attributed the superior performance to hydrophilicity, good electrical conductivity, and ability of the 1T MoS_2_ to expand to host the intercalating ions [102]. Also, it has been demonstrated that some of the 2D TMDs, e.g., MoS_2_ and WS_2_, undergo conversion reactions, especially when interacting with Na^+^ ions [103].

#### 2D TMD Anodes for Non-Li Metal Ion Rechargeable Batteries

2D TMDs have been used as anode materials in rechargeable non-Li metal ion batteries, and the highlights of some notable performance characteristics are detailed in this section.

Ab initio studies of TMDs, primarily considering of MoS_2_ as the template anode, have been performed to elucidate the mechanism of alkali ion interaction with the layered structure.

Mortazavi et al. used first-principles method to analyze the intercalation of Na^+^ ions into the MoS_2_ crystal lattice [104]. They demonstrated a phase change from 2H to 1T MoS_2_ with the formation of intermediate Na_*x*_MoS_2_. The 2H phase was stable at all concentrations, whereas the 1T phase was only possible for *x* > 0.25 [104]. The authors have also shown the working potential to be 1.25 V. An open circuit voltage of 2.5–3.5 V is possible if coupled with a suitable positive electrode [104, 105]. Along similar lines, Li et al. performed DFT analysis together with experimental work to investigate the intermediate products formed during the process of Na^+^ ion intercalation into MoS_2_ [106]. They have reported on the presence of several metastable phases during the process, e.g., Na_0.375_MoS_2_, Na_0.625_MoS_2_, Na_1.75_MoS_2_. The phase transitions from 2H when Na content exceeds 0.375, with addition Na intercalation resulting in the rearrangement of the structure to the 1T morphology [106].

The application of *bare TMD nanosheets* as anode materials has been investigated by several groups. Su et al. experimented with few-layered MoS_2_ nanosheets and utilized an exfoliation technique to produce an anode for SIBs. They were able to observe a reversible specific capacity of 386 mAh g^−1^ after 100 cycles of galvanostatic charge-discharge (GCD) at an operating current density of 40 mA g^−1^ [107]. MoS_2_ sheets, exfoliated in organic solvents (1-methyl-2-pyrrolidone), were studied as anodes in SIBs by Bang and coworkers [108]. First-cycle sodiation and desodiation capacities of 254 and 164 mAh g^−1^ were obtained respectively, with a Coulombic efficiency of 97% after 50 cycles [108]. An experimental analysis to study the SEI formation in a SIB with a MoS_2_ nanoflake anode was performed by Lacey et al. [109]. The results indicate that an irreversible change in surface morphology occurs (“permanent wrinkling”) at ~ 0.4 V at the edges and the height of these undulations ranged from 7 to 36 nm [109]. The authors have attributed the undulations or “wrinkling” to the effect of Na^+^ ion intercalation, with further undulations arising due to SEI formation at approximately 1.5 V [109].

WSe_2_, in SIBs, was investigated by Share et al. They were able to obtain a reversible capacity of 200 mAh g^−1^ at a current density of 20 mA g^−1^ [110]. Ex situ results indicate the formation of isolated W and Na_*x*_Se as intermediates during the Na ion intercalation process [110].

**Fig. 5:**
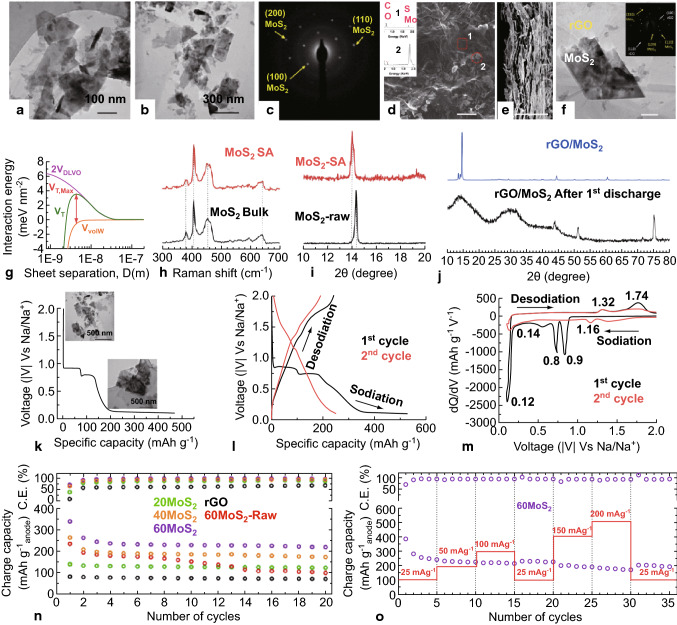
Structural characterization and electrochemical performance analysis of TMD (MoS_2_) as an electrode material in SIB: **a**, **b** HRTEM images of exfoliated MoS_2_ nanosheets obtained after treatment with a superacid. **c** Selected area diffraction pattern for the MoS_2_ nanosheet imaged in Fig. **b**. **d**, **e** SEM images of the top and cross-sectional view of the 60 MoS_2_ sample (60 wt% MoS_2_-40 wt% rGO), respectively. **e** TEM image of SAED pattern (in the inset) of the same 60 MoS_2_ sample. **g** Graphical representation of the total interaction potential (VT), repulsion (*V*_DLVO_), and attraction energy (*V*_vdW_) versus greater sheet separation. **h** Raman spectra of the MoS_2_ samples, in bulk (black) and after exfoliation (red) by acid treatment. **i** XRD spectra of the bulk (black) and exfoliated MoS_2_ (red) sample, indicating the peak shift and the broadening of the consequent increase in the interlayer distance of the exfoliated sample. **j** XRD pattern of pristine MoS_2_-rGO (60 MoS_2_) electrode (blue) and the same after one discharge cycle (black). Considerable peak broadening is observed due to sodiation. **k** First discharge graph of the 60 MoS_2_ electrode, also showing the formation of nanocrsytallites along the process from the TEM images and SAED patterns in the inset. **l** 1st (black)- and 2nd-cycle (red) GCD curves for the 60 MoS2 sample. **m** Differential capacity curves for the first two cycles demonstrating the redox voltages. **n** Charging capacity and Coulombic efficiency values for the different composite and bare rGO sample performed at a current density of 25 mA g^−1^. **o** Charge capacity, coulombic efficiency, and rate capability of 60 MoS_2_ sample [83]. Copyright 2014 American Chemical Society. Scale bar for **d**, **e** is 10 μm and for **f** is 100 nm. (Color figure online)

*TMD composites* have also been used to harness the overall beneficial properties of both materials. A MoS_2_-rGO layered nanocomposite fabricated by a novel, facile acid-based exfoliation technique was investigated as an anode in SIB by David et al. [83]. They were able to achieve a sodiation capacity of 230 mAh g^−1^ at a current density of 25 mA g^−1^ after 20 cycles of operation [83]. A summary of the results obtained by David et al. as representative of the performance of TMD anodes, in general, is presented in Fig. 5.

A MoS_2_/C composite was fabricated by Li et al. utilizing a polymethacrylate-based resin precursor, for application as an anode in SIBs [111]. A first-cycle discharge capacity of 784.3 mAh g^−1^ was achieved at current densities of 50 mA g^−1^ [111]. MoS_2_ nanosheets with a preferred orientation (002 plane) grown on CNTs were studied by Zhang et al. [112]. The authors were able to achieve a specific capacity of 495.9 mAh g^−1^ at a current density of 200 mA g^−1^ with a capacity retention of 84.8% [112]. Probing of the reaction mechanism indicated that the sodiation/desodiation process was driven by a conversion reaction [112]. A novel SIB consisting of exfoliated MoS_2_ nanosheets reinforced with single-walled carbon nanotubes (SWNTs) resulted in a specific capacity of approximately 420 mAh g^−1^ and only 5% capacity loss after 100 cycles of operation [113]. The authors have attributed the superior performance to the formation of a composite which greatly improved the conductivity and formed a strong network that was deemed to be resistant to mechanical failure [113].

A layered MoSe_2_ nanosheet structure supported by multi-walled carbon nanotubes (MWNTs) was studied by Zhang et al. as an anode in SIB [114]. A reversible specific capacity of 459 mAh g^−1^ was obtained at a current density of 200 mA g^−1^ after 90 cycles of operation [114]. The authors have proposed that a “synergistic” influence of the composites helped to produce a net improved performance compared to the individual components [114]. A MoSe_2_ nanosheet/C cloth composite has been investigated as a flexible anode in SIBs and yielded an initial capacity as high as 452.6 mAh g^−1^ [115]. Some loss occurs in the initial cycles due to SEI formation, and 85.5% of the initial capacity is retained after 100 cycles at a current density of 0.2 A g^−1^. The composite performs better than the bare MoSe_2_, whose poor performance has been attributed to a lack of enhanced conducting pathways and a smaller active surface area [115]. Niu et al. studied MoSe_2_ grown on N- and P-doped C nanosheets via a solvothermal reaction [116]. A specific capacity of 378 mAh g^−1^ was achieved after 1000 cycles of operation at a current density of 0.5 A g^−1^, and this corresponded to a capacity retention of 87% [116]. The authors indicated that doping of the elements into the composite assisted in improving the charge transfer kinetics of the overall system [116].

TMDs have been studied as anodes in other metal ion rechargeable systems as well, albeit sparingly. A vertical MoS_2_ “nanorose” grown on graphene has been investigated as an anode for application in KIB [117]. The authors reported relatively high specific capacities of 679 mAh g^−1^ at a current density of 20 mA g^−1^. The high specific capacities were attributed to the increase in the interlayer spacing of the MoS_2_ as a result of its rose pattern and composite formation with graphene [117]. WSe_2_ for Mg ion systems has been studied by Liu and coworkers [118]. They reported a reversible specific capacity of 120 mAh g^−1^ at a high current density of 1500 mA g^−1^ with a retention of approximately 83.5% after 50 cycles [118].

A summary of the works highlighted in this section and other notable works on the application of layered 2D TMD anodes in emerging metal ion rechargeable battery systems are presented in Table 2.

**Table 2:** Performance of layered TMD anode, in various non-Li metal ion rechargeable battery systems

Type of TMD anode	Electrolyte chemistry	Voltage range (V)	Performance*	References
MoS_2_ nanosheets	1 M NaClO_4_ in EC/PC (1:1)	0.01–3.00	386/100/40	[107]
MoS_2_ nanosheets	1 M NaClO_4_ in FEC/PC (1:1)	0.4–2.60	161/50/0.02C^a^	[108]
WSe_2_	1 M NaPF_6_ in PC/EC/DEC (1:1:0.05)	0.10–2.50	117/30/0.1C^a^	[110]
MoS_2_/graphene sheets	1 M NaClO_4_ in DMC/EC (1:1)	0.1–2.25	218/20/25	[83]
MoS_2_/C nanosheets	1 M NaClO_4_ in EC/PC/FEC (1:1:0.05)	0.1–3.0	418.8/50/50	[111]
MoS_2_/SWNT	1 M NaClO_4_ in EC/DEC (1:1)	0.1–3.0	390/100/100	[113]
MoSe_2_/MWNT	1 M NaClO_4_ in EC/DMC/FEC (1:1:0.05)	0.1–3.0	459/90/200	[114]
MoSe_2_/C cloth composite	1 M NaClO_4_ in EC/DMC/FEC (1:1:0.05)	0.0–3.0	386.9/100/200	[115]
MoS_2_/graphene “nanorose”	0.8 KPF_6_ in PC/EC/FEC (1:1:0.02)	0.01–3.00	381/100/100	[117]
MoS_2_/C nanosheets	1 M NaClO_4_ in EC/DEC/PC (1:1:1)	0.01–2.90	280/300/1C^a^	[119]
MoS_2_/PEO nanocomposites	[Mg_2_Cl_3_]^+^[AlCl_2_Ph_2_]^-^	0.2–2.00	75.2/30/5	[120]
MoSe_2_ nanoplates	1 M NaClO_4_ in PC	0.1–3.00	369/50/0.1C	[121]
WSe_2_/C	1 M NaPF_6_ in EC/DMC/FEC (1:1:0.05)	0.01–3.00	270/50/0.2C^a^	[122]
Layered MoS_2_	0.5 M KPF_6_ in PC/EC (1:1)	0.50–2.00	65/200/50	[123]
Layered MoS_2_/rGO	AlCl_3_-PhMgCl	0.01–2.10	80/50/20	[124]

*Specific capacity (mAh g^−1^)/number of cycles/current density (mA g^−1^)

^a^C rate

#### 2D TMD Electrodes for Non-Li Supercapacitors

TMDs have been applied in (aqueous) supercapacitors as well. A few prominent results will be examined in this section.

Layered MoS_2_ obtained by a microwave-based heating technique loaded on rGO for support has been investigated by Firmiano and coworkers in supercapacitors [125]. A maximum specific capacitance of 265 F g^−1^ was obtained at a scan rate of 10 mV s^−1^ with an energy density of 63 Wh kg^−1^ [125]. A layered Mos_2_-graphene composite as a supercapacitor electrode was studied by Huang et al. [126]. A maximum specific capacitance of 243 F g^−1^ was reported by the authors in addition to an energy density of 73.5 Wh kg^−1^ [126]. The authors have attributed the observed cycling stability to efficient charge transport through the composite as well as its ability to prevent large volume expansion [126]. MoS_2_/polyaniline (PANI) nanosheet composites prepared by an aniline-based intercalation process were investigated as a supercapacitor electrode by Wang et al. [127]. The composite electrode provided a specific capacitance of 390 F g^−1^ and a capacity retention of 88.6% over 1000 cycles. These composites perform significantly better than the bare PANI sample (131 F g^−1^ specific capacitance and 42% retention over 600 cycles) [127]. The authors attributed the superior result of the composite to the nanocomposite architecture which enhanced net cycling stability and conductivity [127].

**Fig. 6:**
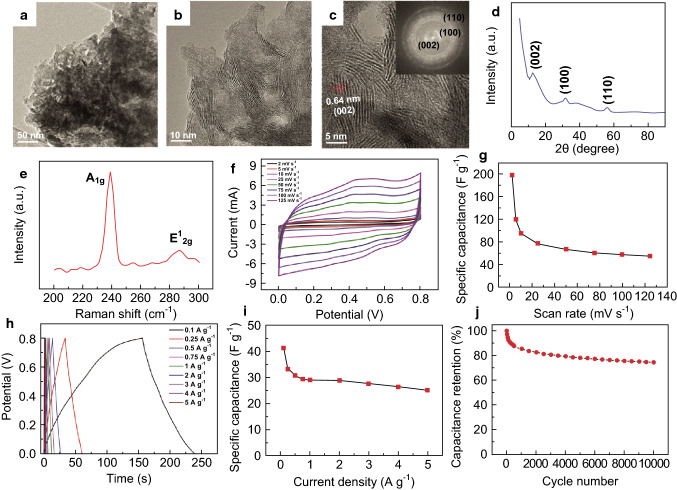
Structural characterization and electrochemical performance analysis of TMD (MoSe_2_) as a supercapacitor electrode: **a**, **b** TEM images of MoSe_2_ nanosheets at low magnification. **c** HRTEM image of the MoSe_2_ sheets with the FFT pattern demonstrated in the inset. **d** XRD spectra of the few-layered MoSe_2_ nanosheets showing the characteristic peaks. **e** Characteristic Raman spectra of the few-layered MoSe_2_ nanosheets where the A_1g_ and ${\text{E}}_{{2{\text{g}}}}^{1}$ vibration modes are clearly visible. **f** CV of the few-layered MoSe_2_ sample in 0.5 M H_2_SO_4_ with scan rates varying from 2 to 125 mV s^−1^. **g** Corresponding specific capacitance values at the given scan rates. **h** First-cycle GCD curves at different current rates, of the few-layered MoSe_2_ sample. **i** Specific capacitance values for the corresponding current rates. **j** Specific capacitance retention of the few-layered MoSe_2_ sample, being run for 10,000 cycles at a current rate of 5 A g^−1^ in 0.5 M H_2_SO_4_ electrolyte [129]. Copyright 2015 Royal Society of Chemistry

Micro-supercapacitors are a relatively novel field of study. In this regard, Cao et al. designed a micron-scale supercapacitor with a thin film of layered MoS_2_ as an active electrode material [128]. MoS_2_ nanosheets were spray-painted on a SiO_2_ substrate, and then laser patterning was performed to obtain the thin films. A volumetric capacitance of 178 F cm^−3^ and aerial capacitance of 8 mF cm^−2^ were obtained, which present reasonable performance [128]. Few-layered MoSe_2_, prepared by a hydrothermal technique, was studied as a supercapacitor electrode by Balasingam et al. [129]. A maximum specific capacitance of 198.9 F g^−1^ was obtained with a capacitance retention of 75% after 10,000 cycles of operation [129]. Figure 6 represents a summary of the results of this group as representative of the performance of TMDs as a supercapacitor electrode.

Huang et al. studied MoSe_2_ nanosheets on Ni foam for supercapacitor applications [130]. A very high capacity of 1114. 3 F g^−1^ with almost 100% capacitance retention after 1500 cycles was obtained [130]. A MoSe_2_/graphene composite on Ni foam as a supercapacitor electrode was analyzed by Huang et al. The composite exhibited a specific capacity as high as 1422 F g^−1^ at a current density of 5 A g^−1^, and 100% of the specific capacitance was retained after 1500 cycles [131]. These excellent properties were attributed to the large surface area of the composite and improvement in its electrical conductivity as well as kinetics [131].

WS_2_, exfoliated by tert-butyllithium (t-Bu-Li), has shown promise as a supercapacitor material [132]. A gravimetric capacitance of 34 F g^−1^ was obtained when compared with MoS_2_, MoSe_2_, and WSe_2_, which were exfoliated using the same technique [132]. This result also shows the importance of the exfoliating medium. Specifically, when WS_2_ was exfoliated in a methyllithium (Me-Li) media, it exhibited a capacitance of only approximately 4 F g^−1^ [132]. Layered WS_2_-rGO composite as a supercapacitor electrode material in aqueous 1 M Na_2_SO_4_ solution has been studied by Ratha and Rout [133]. A specific capacitance of 350 F g^−1^ was achieved at a scan rate of 2 mV s^−1^, and the composite performed better than individual WS_2_ and rGO nanosheets [133]. Table 3 gives a summary of the performance of the aforementioned layered TMDs used in supercapacitors.

**Table 3:** Performance of layered 2D TMD electrodes in various non-Li supercapacitor systems

Type of TMD	Electrolyte chemistry	Performance*	References
MoS_2_/rGO	1 M HClO_4_	265 F g^−1^/10 mV s^−1^	[125]
MoS_2_/graphene	1 M Na_2_SO_4_	243 F g^−1^/1 A g^−1^	[126]
MoS_2_	1 M NaOH	178 F cm^−3^/10 mV s^−1^	[127]
MoS_2_/PANI	1 M H_2_SO_4_	390 F g^−1^/0.8 A g^−1^	[128]
MoSe_2_	0.5 M H_2_SO_4_	199 F g^−1^/2 mV s^−1^	[129]
MoSe_2_	6 M KOH	1114.3 F g^−1^/1 A g^−1^	[130]
MoSe_2_/graphene	6 M KOH	1422 F g^−1^/5 A g^−1^	[131]
WS_2_	0.1 M 7.4 pH PBS	40 F g^−1^/0.5 A g^−1^	[132]

*Specific capacitance (F g^−1^)/scan rate (mV s^−1^)

### Electrochemistry of MXenes

MXenes presents a low diffusion barrier for intercalation of various alkali metal ions between their sheets and therefore is not suitable for application as negative electrode materials in non-Li ion rechargeable battery systems [48]. However, depending on the fabrication technique and the type of terminating agent, various MXenes can function in a range of potential windows, thereby providing considerable flexibility in terms of their performance [48]. It has been observed that MXenes of the general formula M_2_X tend to demonstrate greater specific capacities than the ones with their corresponding higher molecular weight counterparts such as M_3_X_2_ and M_4_X_3_, since the low-formula-weight MXenes can store a greater number of ions [134]. The presence of the terminating functional group has been demonstrated to significantly affect the net specific capacity exhibited by the MXene, e.g., MXenes with oxygen-containing terminating groups exhibit much larger specific capacity than those with fluorine-containing groups [135]. The addition of intercalating particles between the MXene sheets has been shown to greatly enhance their specific capacity and electrode longevity [136].

#### MXene Anodes for Non-Li Metal Ion Rechargeable Batteries

*A first*-*principles study* of M_2_C-type MXene (where *M* = transition metal such as Ti, V, Cr etc.) as a Na-ion battery anode was performed by Yang et al. using density functional theory (DFT) [65]. The results revealed a low anode voltage (< 1.5 V) and activation energy (0.09–0.18 eV) for Na ion migration in MXenes which suggests a high energy density and high rate capability [65]. Along similar lines, the intercalation of different alkali ions (Na, K and Ca) into Ti_3_C_2_ were studied by Er et al. Theoretical specific capacities of 351.8, 319.8, and 191.8 mAh g^−1^ were obtained for Na, Ca and K, respectively, and reported by the authors [137]. Theoretical DFT-based studies on the applicability of Mo_2_C monolayers have been recently performed by Sun and coworkers [138]. They have demonstrated diffusion barriers of 0.0035 and 0.0015 eV for Li and Na atoms, respectively, into the Mo_2_C monolayer lattice, indicating the probability of good diffusion and superior charge and discharge rates [138].

Experimental results for the electrochemical performance of MXene (Ti_3_CNT_*x*_) as an anode material was reported by Naguib et al. for the K-ion battery [139]. For the first cycle, 202 mAh g^−1^ capacity recovered was achieved (with 28.4% coulombic efficiency, 1.5 K^+^ correlation) which continuously degraded to 75 mAh g^−1^ after 100 cycles (with 0.6 K^+^ correlation) [139]. Natu et al. studied the influence of pH variation during fabrication of 2D MXenes [140]. Crumpled mesoporous MXene (Ti_3_C_2_T_*x*_) flakes were synthesized via a reduction in the pH of the MXene suspension. A sodium ion battery using this electrode as an anode resulted in specific capacities of 250 and 120 mAh g^−1^ at 20 and 500 mA g^−1^, respectively [140]. In order to avoid restacking behavior and to improve the limited capacity of bare MXene, Wu et al. recently proposed an efficient technique which involved the insertion of SnS_2_ nanoplates between MXenes (Ti_3_C_2_T_*x*_) interlayers [141]. The MXene/SnS_2_ composites were synthesized by self-assembly of sonicated MXene suspension and hydrothermal-synthesized SnS_2_. Electrochemical measurements revealed a capacity retention of 120 mAh g^−1^ for a 100 mA g^−1^ current density after 125 cycles at 0 °C for a MXene/SnS_2_ 5:1 (mass ratio) electrode [141]. In another investigation by the same group, MoS_2_ nanosheets were used as intercalation particles between MXene (Ti_3_C_2_T_*x*_) layers [142]. The MXene/MoS_2_ composite resulted in a reversible capacity of 250.9 mAh g^−1^ after 100 cycles, while 162.7 mAh g^−1^ could be retained as the capacity at 1 A g^−1^, indicating a high rate capability [142]. Similarly, a MXene/Sb_2_O_3_ composite material was developed by Guo et al. through a facile solution phase fabrication technique [143]. The specific capacities of the as-prepared composite material as anodes for Na-ion batteries were measured to be as high as 472 mAh g^−1^ at 100 mA g^−1^ after 100 cycles. It also exhibited a high rate capability of 295 mAh g^−1^ at 2 A g^−1^ as well [143].

**Fig. 7:**
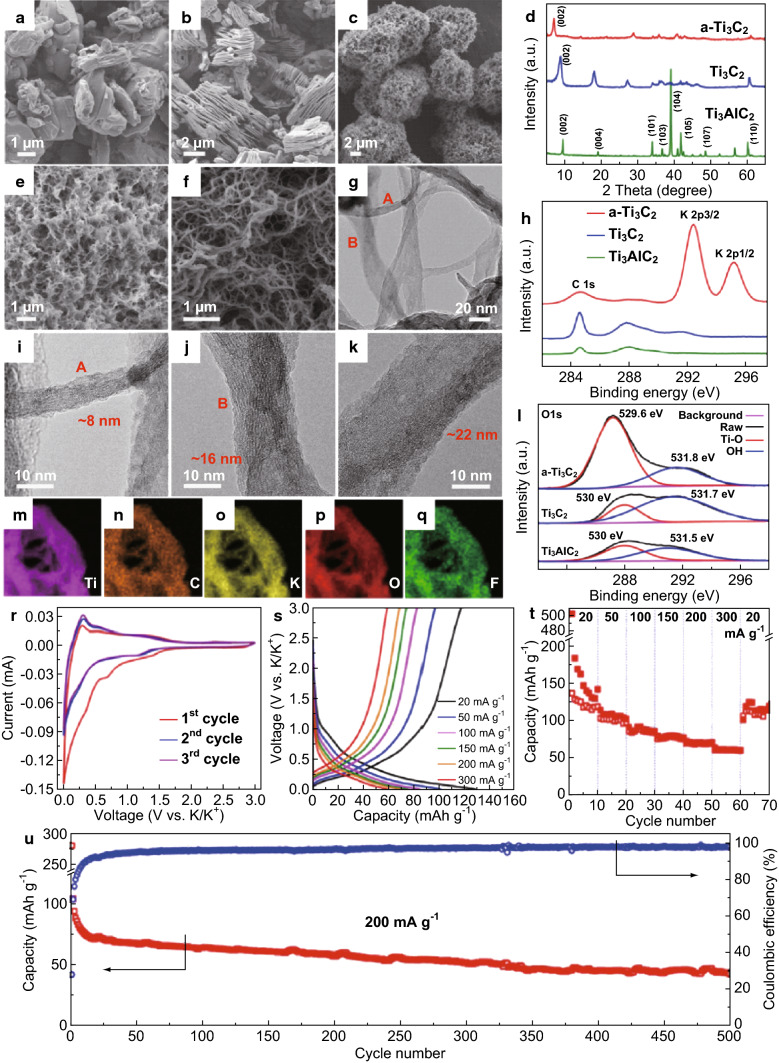
Structural characterization and electrochemical performance analysis of MXene as electrode material in KIB: **a**–**c** SEM images of Ti_3_AlC_2_ MAX phase, Ti_3_C_2_ MXene nanosheets (MNSs) obtained by etching the precursor MAX phase with 40% HF solution and KOH-treated Ti_3_C_2_ MNSs (a-Ti_3_C_2_), respectively. **d** XRD spectra stack of Ti_3_AlC_2_, Ti_3_C_2_ and a-Ti_3_C_2_. **e** SEM image of Ti_3_C_2_ MXene nanoribbons (MNRs) at low magnification. **f** Corresponding image at high-magnification **g** TEM image of MNRs h XPS spectra of Ti_3_AlC_2_, Ti_3_C_2_, and a-Ti_3_C_2_ showing the free C and K peaks. **i**–**k** HRTEM images of the Ti_3_C_2_ MNRs at different positions (“A” and “B”) and varying thicknesses (8, 16, and 22 nm). **l** XPS spectra of the 3 samples showing the O 1s peak. **m**–**q** Elemental mapping of Ti, C, K, O and F regions of the a-Ti_3_C_2_ sample, respectively. **r** CV of the a-Ti_3_C_2_ MNRs for KIB collected at a scan rate of 0.1 mV s^−1^. **s** GCD curves of the sample obtained at different given current rates. **t** Rate capability of the a-Ti_3_C_2_ sample. **u** Cycling performance at 200 mA g^−1^ and coulombic efficiency for the a-Ti_3_C_2_ sample [145]. Copyright 2018 Elsevier

MXenes (Ti_3_C_2_) have also been used as precursors to fabricate Na or K containing titanate nanoribbons as anodes by Dong et al. under the alkalization effect of NaOH or KOH [144]. These ultrathin ribbons presented 191 mAh g^−1^ (Na titanate) for the Na-ion battery at 200 mA g^−1^. Likewise, 151 mAh g^−1^ (K titanate) was achieved for the K-ion battery at 50 mA g^−1^ [144]. Lian et al. studied an alkalized Ti_3_C_2_ MXene nanoribbon for SIB and KIB systems [145]. The MXene produced a specific capacity of 42 mAh g^−1^ for the KIB system after 500 cycles of operation and the authors have attributed the superior results to the enhanced interlayer spacing which allows for storage of more ions [145]. A summary of Lian’s results is presented in Fig. 7, which is representative of the performance of MXene anodes in KIB systems.

Table 4 provides a list of the various notable MXene anode performances in emerging metal ion rechargeable battery systems.

**Table 4:** Performance of MXene electrodes, in various non-Li metal ion rechargeable battery systems

Type of MXene	Electrolyte chemistry	Voltage range (V)	Performance*	References
Ti_3_CNT_*x*_	0.8 M KPF_6_ in EC/DEC (1:1)	0.005–3	75/100/20	[139]
Ti_3_C_2_T_*x*_	1 M NaClO_4_ in EC/PC/FEC (1:1:0.05)	0.001–3	246/50/20	[140]
Ti_3_C_2_T_*x*_	1 M NaClO_4_ in EC/PC (1:1)	0.01–2.5	322/200/100	[141]
Ti_3_C_2_T_*x*_	1 M NaPF_6_ in EC/DEC (1:1)	0.1–3	100/100/20	[146]
Ti_3_C_2_T_*x*_/MoS_2_	1 M NaClO_4_ in EC/PC (1:1)	0.01–3	250.9/100/100	[142]
Ti_3_C_2_T_*x*_/Sb_2_O_3_	1 M NaClO_4_ in EC/PC (1:1)	0.01–2.5	472/100/100	[143]
Ti_3_C_2_ ribbon	1 M NaCF_3_SO_3_	0.01–3	113/200/50	[145]
Ti_3_C_2_ ribbon	0.8 M KPF_6_	0.01–3	42/500/200	[145]
Ti_3_C_2_T_*x*_/CTAB	0.4 (PhMgCl)_2_-AlCl_3_/THF	0.01–2	135^a^/250/20	[147]
Ti_3_C_2_T_*x*_/CNT	1 M NaClO_4_ in EC/PC/FEC (1:1:0.05)	0.01–3	164/100/50	[148]
Sn-Ti_3_C_2_	1 M LiPF_6_ in EC/PC (1:1)	0.01–3	500/200/500	[136]

*Specific capacity (mAh g^−1^)/number of cycles/current density (mA g^−1^)

^a^Volumetric capacity (mAh cm^−3^)

#### MXene Electrodes for Non-Li Supercapacitors

**Fig. 8:**
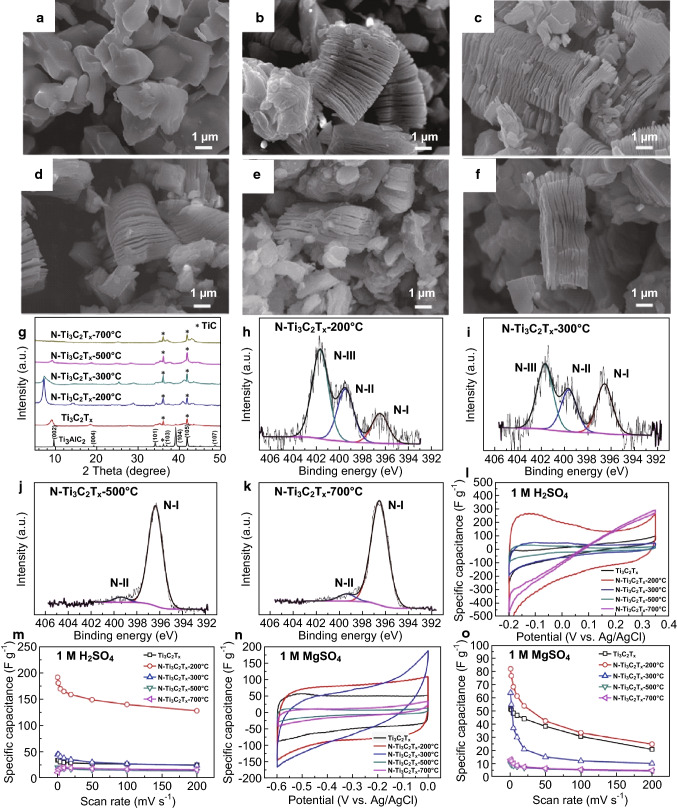
Structural characterization and electrochemical performance analysis of MXene (N-doped Ti_3_C_2_T_*x*_) as a supercapacitor electrode: SEM images of **a** Parent MAX phase Ti_3_AlC_2_. **b** Ti_3_C_2_T_*x*_ obtained after etching the MAX phase with HF. **c** N-doped Ti_3_C_2_Tx synthesized at 200 °C (N–Ti_3_C_2_T_*x*_-200 °C). **d** N–Ti_3_C_2_T_*x*_-300 °C. **e** N–Ti_3_C_2_T_*x*_-500 °C. **f** N–Ti_3_C_2_T_*x*_-700 °C. **g** XRD spectra stack of the parent MAX phase (Ti_3_AlC_2_) and the non-doped and various N-doped Ti_3_C_2_T_*x*_ MXenes. **h**–**k** XPS spectra of N–Ti_3_C_2_T_*x*_-200 °C, N–Ti_3_C_2_T_*x*_-300 °C, N–Ti_3_C_2_T_*x*_-500 °C, N–Ti_3_C_2_T_*x*_-700 °C samples, respectively. **l** CV curves of the non-doped and doped MXenes in 1 M H_2_SO_4_ at 1 mV s^−1^ scan rate. **m** Specific capacitance of the MXenes corresponding to the scan rates in **i** and **n** CV curves of the non-doped and doped MXenes in 1 M MgSO_4_ at 1 mV s^−1^ scan rate. **o** Specific capacitance of the MXenes corresponding to the scan rates in **n** [150]. Copyright 2018 Elsevier

MXenes have also been exploited for use as electrodes in supercapacitors due to their excellent cyclability and high capacitance [67]. Xu et al. obtained binder-free MXene (Ti_3_C_2_T_*x*_) nanoflakes using a modified electrophoretic deposition (MEPD) method. The MXene films were then self-assembled on nickel foam. The flexible solid-state supercapacitor using as-prepared MXene electrodes demonstrated a specific capacitance of 140 F g^−1^ in 1 M KOH without capacitance degradation after 10,000 cycles. Ti_3_C_2_ MXene was prepared by Gao et al. from Ti_3_AlC_2_ via selective etching of Al in HF, resulting in a surface area of 22.34 m^2^ g^−1^ [149]. Electrochemical characterization showed a volumetric capacitance of 119.8 F cm^−3^ in 3 M KOH electrolyte at 2.5 A g^−1^ which retained 94.2% (112.9 F cm^−3^) after 1000 cycles. This performance can be conveniently improved by elemental doping according to Wen et al., such as N-doped the procedure of Ti_3_C_2_T_*x*_ which was proposed in their research [150]. Annealing MXene in flowing ammonia at a temperature of 200–700 °C provided concentration-controlled N-Ti_3_C_2_T_*x*_ with nitrogen contamination of 1.7–20.7 at%. A significantly elevated specific capacitance of 192 F g^−1^ was obtained, compared to 34 F g^−1^ from a pure Ti_3_C_2_T_*x*_ anode, in the same 1 M H_2_SO_4_ electrolyte [150]. The main results of this work are shown in Fig. 8 as a representative example of MXene’s performance as a supercapacitor electrode. Another investigation by Wang et al. demonstrated that an even superior specific capacitance of 1061 F g^−1^ at 1 A g^−1^ achieved using (Ti_3_C_2_)/Ni-Al double-layer hydroxide composite with high rate capability achieved a specific capacitance of 556 F g^−1^ at 10 A g^−1^ [151].

Table 5 lists the performance of several notable MXenes which have been used for non-Li supercapacitor applications.

**Table 5:** Performance of MXene electrodes in various non-Li supercapacitor systems

Type of MXene	Electrolyte chemistry	Performance*	References
Ti_3_C_2_T_*x*_ film	1 M KOH	140 F g^−1^/5 mV s^−1^	[67]
Ti_3_C_2_	3 M KOH	119.8 F cm^−3^/2.5 A g^−1^	[149]
N–Ti_3_C_2_T_*x*_	1 M H_2_SO_4_	192 F g^−1^/1 mV s^−1^	[150]
Ti_3_C_2_/Ni–Al	6 M KOH	1061 F g^−1^/1 A g^−1^	[151]
Ti_3_C_2_T_*x*_/rGO	1 M MgSO_4_	435 F cm^−3^/2 mV s^−1^	[152]
Ti_3_C_2_T_*x*_/rGO	3 M H_2_SO_4_	1040 F cm^−3^/2 mV s^−1^	[153]
Ti_3_C_2_T_*x*_ film	EMI-TFSI	70 F g^−1^/20 mV s^−1^	[154]
Ti_2_CT_*x*_–N_2_/H_2_	30 wt. % KOH	51 F g^−1^/1 A g^−1^	[155]
Ti_3_C_2_	1 M KOH	76 F g^−1^/2 mV s^−1^	[156]

*Specific capacitance (F g^−1^)/scan rate (mV s^−1^)

### Electrochemistry of Phosphorene

Phosphorene, single/few layers of black phosphorus has demonstrated a high theoretical specific capacity (2596 mAh g^−1^ for Li^+^ ions); however, large volume expansion on interaction with intercalating ions remains an important concern [157, 158]. Theoretical studies have demonstrated an anisotropic intercalation of Na^+^ ions into phosphorene, which can be attributed to its puckered honeycomb-like structure [159]. Density functional studies have also demonstrated that adsorption of alkali ions, especially Na^+^ ions, usually occurs on both sides of the phosphorene sheet [159]. Since adsorption over adjacent sides reduces the net repulsion, this arrangement is favored over ionic adsorption for a single side [159]. Several intermediate metastable phases have been reported, especially for Na^+^ ion intercalation, e.g., NaP_8_, NaP_2_, and are observed during the cycling of phosphorene anode [26, 160].

#### Phosphorene Anodes for Non-Li metal Ion Rechargeable Batteries

Some notable studies on phosphorene anodes in non-Li metal ion rechargeable batteries are outlined in the following discussion.

Kulish et al. performed a theoretical study of phosphorene for SIB systems and reported a maximum theoretical specific capacity of 865 mAh g^−1^ with a resultant NaP stoichiometry [159]. Layered BP, essentially the precursor of phosphorene, has been studied as an anode in SIBs by Peng and coworkers [161]. The researchers essentially used a BP-carbon black nanocomposite anode were able to achieve 1381 mAh g^−1^ after 100 cycles of operation at a rate of 90.5%. Increased conductivity and the enhanced electroactive surface area of the nanocomposite played a decisive role in facilitating such high capacities, according to the authors [161]. BP-graphitic composite anodes in KIBs have been studied by Sultana et al. The first-cycle specific capacity obtained was 617 mAh g^−1^, which is more than twice that of pristine graphite for SIBs with 61% retention over 60 cycles of operation. The authors proposed an alloying reaction mechanism for the anode. It was determined that the composite with a weight ratio BP/C of 1:1 performed best [162].

**Fig. 9:**
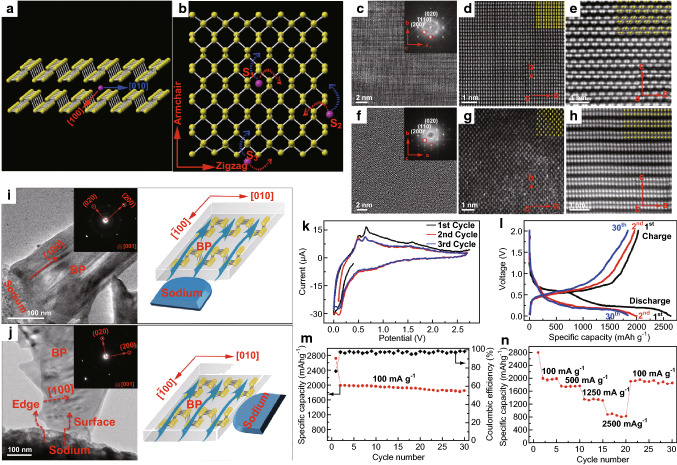
Structural characterization and electrochemical performance analysis of phosphorene electrode material in SIB: **a** Schematic showing the movement of Na^+^ ion between phosphorene layers. **b** Energetics dictated transport of Na^+^ ions in the interstices of phosphorene lattice. **c**, **f** HRTEM images along the [001] direction of few-layer phosphorene and single-layer phosphorene, respectively, with FFTs shown in the inset of each. **d**, **g** Atomic-scale high-angle annular dark field image (HAADF) along the [001] direction of few-layer phosphorene and single-layer phosphorene, respectively, with atomic orientations in inset (in yellow). **e**, **h** HAADF of few-layer phosphorene along the [100] and [010] directions, respectively, with the corresponding atomic orientations in the inset (in yellow). **i**, **j** TEM image and schematic of the contact interface of Na^+^ ion normal to the [100] direction and along the [100] direction, respectively. The SAED patterns of the few-layered phosphorene are shown in the inset of both. **k** CV of the first three cycles of the phosphorene electrode for the half cell at a scan rate of 0.005 mV s^−1^. **l** GCD profiles of the 1st, 2nd, and 30th cycle for the phosphorene electrode in the half cell. **m** Capacity retention and coulombic efficiency of the phosphorene electrode running at a current density of 100 mA g^−1^. **n** Rate capability of the phosphorene anode [163]. Copyright 2016 American Chemical Society. (Color figure online)

Nie et al. reported a maximum discharge capacity of 2631 mAh g^−1^ with a Coulombic efficiency of 77% for phosphorene as a SIB anode [163]. The authors were able to probe the movement of the Na^+^ ion into the phosphorene lattice and have provided a model for this behavior [163]. Figure 9 represents a description of the mechanism and the performance of phosphorene as an anode in SIBs.

A phosphorene-graphene (P/G) hybrid anode in SIB systems has been studied by Sun et al. [164]. The authors have identified a 2-step intercalation-alloying mechanism that helps to produce an elevated specific capacity of 2440 mAh g^−1^ at a current density of 50 mA g^−1^ with a capacity loss of only 15% after 100 cycles of operation [164]. Hexagonal boron nitride (h-BN)-capped phosphorene (h-Bn/Pn) as an anode for SIBs was investigated using ab initio techniques by Chowdhury and coworkers [165]. A reversible specific capacity of 445 mAh g^−1^ was obtained with a binding energy of 2.55 eV for Na^+^ ions in h-BN/Pn [165].

Theoretical studies for phosphorene as anodes in Mg ion batteries have demonstrated a diffusion barrier of 0.08 eV with an alloying reaction mechanism and an Mg_0.5_P intermediate [166].

The performance of notable BP/phosphorene systems as anodes in emerging non-Li metal ion rechargeable batteries is provided in Table 6.

**Table 6:** Performance of BP/phosphorene anode, in various non-Li metal ion rechargeable battery systems

Type of Phosphorene	Electrolyte chemistry	Voltage range	Performance*	References
Phosphorene	N.A.	N.A.	433 for NaP_2_ stoichiometry^a^	[159]
P/C composite layers	1 M NaClO_4_ in EC/PEC/FEC (1:1:0.1)	0.005–1.5 V	1500/100/100	[161]
Layered black phosphorus	0.75 M KPF_6_ in EC/DEC (1:1)	0.01–2.00 V	400/50/50	[162]
P/G hybrid	1 M NaPF_6_ in EC/DEC/FEC (1:1:0.1)	0.00–1.50 V	2400/100/0.02C^b^	[164]
Layered black phosphorus	1 M NaPF_6_ in EC/DEC (1:1)	0.00–2.00 V	1500/25/125	[167]
P/G hybrid layers	N.A.	Sodiation between 0.29 and 0.70 V	372^b^	[168]
Phosphorene	N.A.	N.A.	410 for 50% Mg intercalation^a^	[169]
Phosphorene	N.A.	N.A.	310.71 for Mg_0.5_P^a^	[26]

*Specific capacity (mAh g^−1^)/number of cycles/current density (mA g^−1^)

^a^Specific capacity (mAh g^−1^) for theoretical study

^b^C rate

#### BP/Phosphorene Electrodes for Non-Li Supercapacitors

A composite of BP nanoflakes/PANI in pseudocapacitors has been investigated by Sajedi-Moghaddam et al. [170]. A high specific capacitance of 354 F g^−1^ was achieved by the nanocomposite, at a current density of 0.3 A g^−1^. According to the authors, the large surface area generated by the layered BP allowed the PANI to nucleate which produced a greater number of ion channels facilitating enhanced charge storage [170].

Exfoliated BP nanoflakes have been investigated in all-solid-state supercapacitors by Hao et al. [171]. A net stack capacitance of 45.8 F g^−1^ was obtained at a scan rate of 0.01 V s^−1^ with a capacitance retention of 71.8% after 30,000 cycles of operation [171]. Along the same lines, Yang and coworkers used a composite of BP nanoflake/CNT paper as an all-solid-state supercapacitor [172]. A maximum volumetric capacitance of 41.1 F cm^−3^ was obtained at a voltage scan rate of 0.005 V s^−1^, whereas a power density of 821.62 W cm^−3^ was obtained at a scan rate of 500 V s^−1^, indicating its robust performance [172]. A nanocomposite with a BP/CNT weight ratio of 1:4 provided the best results [172]. Luo et al. studied a polypyrrole/BP-laminated film composite as an electrode in a flexible solid-state supercapacitor and obtained a maximum specific capacitance of 497.5 F g^−1^ and almost 100% capacitance retention after 10,000 cycles at a current density of 5 A g^−1^ [173]. The authors attributed the superior performance to the lamination which provided an easier path for the ions and also prevented structural degradation during cycling [173].

**Fig. 10:**
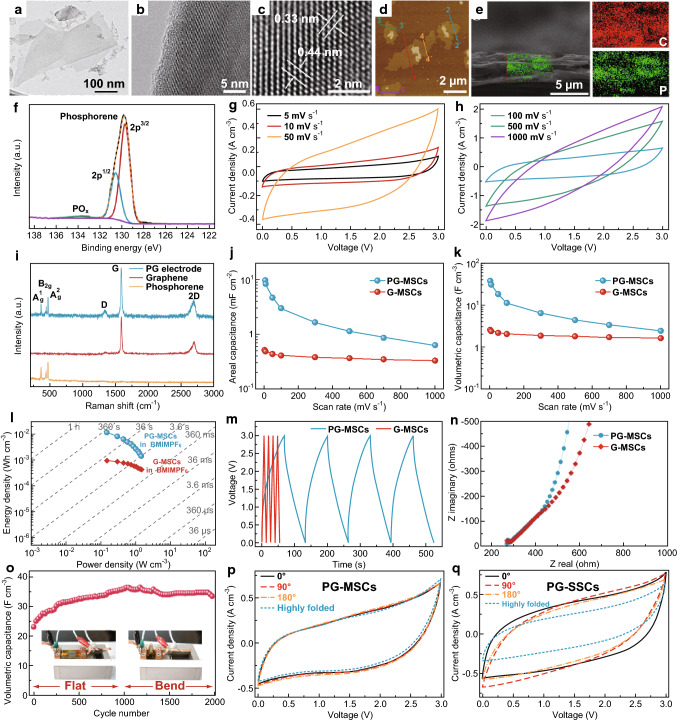
Structural characterization and electrochemical performance analysis of phosphorene as a supercapacitor electrode: **a** TEM image of phosphorene thin film **b** HRTEM image of the phosphorene thin film. **c** HRTEM micrograph of the phosphorene thin film with atomic positions and lattice parameters. **d** AFM image of the phosphorene thin film. **e** SEM image of the cross section of the phosphorene thin film with EDX spectra of the selected region. **f** XPS spectra of phosphorene thin film. **g**, **h** CV at varying scan rates of P/G anode and G anode, respectively. **i** XRD spectra stack of the phosphorene (orange), graphene (red) ,and P/G composite electrode (blue). **j** Aerial capacitances of P/G (blue) and G (red) samples. **k** Volumetric capacitances of P/G (blue) and G (red) samples. **l** Ragone plots of P/G (blue) and G (red) samples. **m** GCD curves of the P/G (blue) and G (red) samples at a current density of 0.3 A cm^−3^. **n** Complex part of the impedance spectra of P/G (blue) and the G (red) samples. **o** Stability performance of the P/G sample over 2000 cycles with the inset pictures showing the flat and bending orientations. **p** CV curves of the P/G composite micro-supercapacitor. **q** CV curves of the P/G composite on PET substrate [174]. Copyright 2017 American Chemical Society. (Color figure online)

Scientific research on phosphorene as an electrode in supercapacitors has been sparingly performed. As a notable exception, Xiao et al. studied a phosphorene-graphene (P/G) interdigital micro-supercapacitor in an ionic liquid environment [174]. A conductivity of 319 S cm^−1^ and an energy density value of 11.6 mWh cm^−3^ were reported by the authors [174]. Figure 10 represents a summary of their results, as representative of the application of phosphorene in non-Li supercapacitors. Flexible double electrodes of phosphorene for supercapacitor applications have resulted in a specific capacitance of 13.75 F cm^−2^ and an energy density of 2.47 mWh cm^−3^ for a scan rate of 0.01 V s^−1^ [175]. A tabular listing of the performance of BP/phosphorene electrodes as non-Li supercapacitors is provided in Table 7.

**Table 7:** Performance of BP/phosphorene electrodes in various non-Li supercapacitor systems

Type of BP/phosphorene	Electrolyte chemistry	Performance*	References
phosphorene/graphene	BMIMPF_6_	9.8 mF cm^−2^/0.44 A cm^−3^	[174]
BP/PANI	1 M H_2_SO_4_	354 F g^−1^/0.3 A g^−1^	[170]
BP nanoflake/CNT	PVA/H_3_PO_4_	41.4 F cm^−3^/5 mV s^−1^	[172]
BP nanosheet/PPy	PVA/H_3_PO_4_	452.8 F g^−1^/0.5 A g^−1^	[173]
BP nanoflake	PVA/H_3_PO_4_	45.8 F g^−1^/10 mV s^−1^	[171]

*Capacitance (areal/volumetric/specific)/current density

## Comparison, Challenges, and the Path Forward

This review has examined the novel properties of 2D materials and illustrated some of their notable applications as rechargeable non-Li metal ion battery anodes and supercapacitor electrodes. However, there are also many challenges associated with the use of 2D materials and important research is being pursued to address them. A few of these limitations will be briefly summarized in the subsequent paragraphs in this section regarding each class of 2D material discussed in this article.

*TMDs* have received the most attention among the various 2D materials reviewed. MoS_2_, the foremost among them, has an interlayer spacing (0.65 nm) large enough to facilitate facile intercalation of Na^+^, K^+^ ions. It also provides a high theoretical capacity (670 mAh g^−1^) for sodium storage [176]. In practice, capacities as high as 530 mAh g^−1^ have been obtained for standalone MoS_2_ systems [107]. However, it is observed that the intercalation of large monovalent alkali metals (e.g., Na^+^, K^+^) into its constituent layers cause significant structural change [177]. This, coupled with the fact that these materials tend to undergo conversion reactions (especially with Na^+^ ionic intercalation), results in the formation of soluble polysulfides which might have undesirable side reactions with the electrolyte [177]. Several techniques have been considered to address these issues such as nanocrystallization, electrode modification, carbon modification. Among these techniques, carbon modification of the MX_2_ TMDs has been studied most exhaustively and it includes the creation of a composite of the TMD with carbon nanotubes, carbon nanoparticles, etc. [177, 178]. This helps to improve porosity, reduce diffusion pathways, and provide a stable support [177, 178]. Controlling the working voltage to prevent the formation of undesirable side products is another technique that has been investigated [177]. In the case of selenides, another issue which has been investigated is cycling stability [179]. To overcome these shortcomings, nanocomposites with carbon-based materials or doping of the selenides have been proposed [179]. Restacking of the TMD sheets, especially for IT-MoS_2_, is an issue that is frequently encountered [180]. Spacers in the form of graphene or carbon nanotubes are introduced between the TMD sheets, and the hybrid structure also has enhanced kinetics [181]. The deadweight of the binder and the current collector are undesirable additives that tend to reduce the gravimetric and volumetric capacitance. To overcome this problem, free-standing electrodes have been studied [83].

*MXenes* too suffer from similar problems of irreversible aggregation and stacking. The incorporation of graphene or conducting polymers between the interlayers has been demonstrated to prevent this problem to a large extent [13]. The use of MXenes in organic electrolytes also poses some challenges, which can also be rectified using conducting spacers, e.g., carbon nanotubes to improve the kinetics and rate performance [48]. Recently, MXene electrodes have also demonstrated notable performance in ionic liquid electrolytes [48, 154]. Another important challenge is to produce MXenes with no surface functional groups. CVD techniques have been proposed to achieve this objective [48].

As anodes in non-Li metal ion rechargeable systems, *Phosphorene* has been applied relatively sparsely compared to TMDs and MXenes. In this case, the scalability of the fabrication procedure is an important issue. The mechanical cleavage technique used is not suitable for bulk production, although it results in highly pure crystals [182, 183]. To address this challenge, plasma-assisted and liquid phase exfoliation processes have been proposed [183]. Also, a large anisotropic volume expansion (~ 92% and ~ 160% along the *y* and *z*-directions, respectively) is observed in pure phosphorene due to Na^+^ ion intercalation, which has been addressed by using it as a nanocomposite with graphene [164].

## Conclusion

Newer and cleaner energy generation techniques require improved and reliable energy storage systems with viable economics. Non-LIBs, e.g., SIBs, KIBs, MIBs, are seen as increasingly attractive alternative because they can be suitably utilized for large-scale grid storage at a reduced cost compared to LIB systems. However, these systems are faced with their own set of challenges which have not been resolved by utilizing conventional anodes analogous to those used in LIB systems.

It is anticipated that 2D materials, with their layered morphology (and consequently large surface area), high mechanical and chemical stability, and their diverse and tunable electronic properties will be gradually introduced as ideal alternatives once the aforementioned limitations are addressed. Moreover, they are best suited to address the highlighted deficiencies of LIB systems. Phenomenologically, the layered structure of these 2D materials will help in accommodating the large mono- and multivalent alkali metal ions and also provide a large number of electroactive sites for reactions to proceed. All these advantages notwithstanding, 2D materials pose their own problems as well; high cost, volume changes during the intercalation process and difficulty in maintaining the sheet-like morphology, poor reversibility, etc., are some of the issues that need to be thoroughly examined in detail before 2D materials can be widely applied. This multitude of fundamental and experimental hurdles can be overcome using a variety of approaches such as the addition of intercalants/dopants to enhance interstitial spacing, the synthesis of composite electrodes to enhance strength, and the manipulation of surface properties via surface functionalization are some of the innovative techniques that are being increasingly exploited to maximize the efficiency of these materials. In addition, these techniques could serve to improve our understanding of the overall electrochemical mechanisms involved, which may provide better insight into efficacious electrode designs which would facilitate the effective exploitation of the capabilities of 2D materials.
